# A Novel PSMA-Targeted Probe for NIRF-Guided Surgery and Photodynamic Therapy: Synthesis and Preclinical Validation

**DOI:** 10.3390/ijms232112878

**Published:** 2022-10-25

**Authors:** Martina Capozza, Rachele Stefania, Valentina Dinatale, Valeria Bitonto, Laura Conti, Cristina Grange, Renata Skovronova, Enzo Terreno

**Affiliations:** 1 Molecular & Preclinical Imaging Centers, Department of Molecular Biotechnology and Health Sciences, University of Torino, 10126 Torino, Italy; 2 Molecular Biotechnology Center, Department of Medical Science, University of Torino, 10126 Torino, Italy; 3 Molecular Biotechnology Center, Department of Molecular Biotechnology and Health Sciences, University of Torino, 10126 Torino, Italy

**Keywords:** prostate cancer, NIRF imaging, photodynamic therapy, image-guided surgery, PSMA, extracellular vesicles

## Abstract

A total of 20% to 50% of prostate cancer (PCa) patients leave the surgery room with positive tumour margins. The intraoperative combination of fluorescence guided surgery (FGS) and photodynamic therapy (PDT) may be very helpful for improving tumour margin delineation and cancer therapy. PSMA is a transmembrane protein overexpressed in 90–100% of PCa cells. The goal of this work is the development of a PSMA-targeted Near InfraRed Fluorescent probe to offer the surgeon a valuable intraoperative tool for allowing a complete tumour removal, implemented with the possibility of using PDT to kill the eventual not resected cancer cells. PSMA-617 binding motif was conjugated to IRDye700DX-NHS and the conjugation did not affect the photophysical characteristics of the fluorophore. The affinity of IRDye700DX-PSMA-617 towards PCa cells followed the order of their PSMA expression, i.e., PC3-PIP > LNCaP > PC3, PC3-FLU. NIRF imaging showed a significant PC3-PIP tumour uptake after the injection of 1 or 5 nmol with a maximum tumour-to-muscle ratio (ca. 60) observed for both doses 24 h post-injection. Importantly, urine, healthy prostate, and the bladder were not fluorescent at 24 h post-injection. Flow cytometry and confocal images highlighted a co-localization of PSMA+ cells with IRDye700DX-PSMA uptake. Very interestingly, ex vivo analysis on a tumour specimen highlighted a significant PSMA expression by tumour-associated macrophages, likely attributable to extracellular vesicles secreted by the PSMA(+) tumour cells. FGS proved that IRDye700DX-PSMA was able to easily delineate tumour margins. PDT experiments showed a concentration-dependent decrease in cell viability (from 75% at 10 nM to 12% at 500 nM), whereas controls did not show any cytotoxicity. PC3-PIP tumour-bearing mice subjected to photodynamic therapy showed a delayed tumour growth. In conclusion, a novel PSMA-targeted NIRF dye with dual imaging-PDT capabilities was synthesized and displayed superior specificity compared to other small PSMA targeted molecules.

## 1. Introduction

Prostate cancer (PCa) is a highly impacting medical challenge affecting a sizable portion of the male population. According to the American Cancer Society, ca. 268490 new cases were expected to be diagnosed in the United States during 2022, with mortality over 10%. PCa will be the most diagnosed cancer in men, representing 26% of the entire cancer diagnosis in the male population [1]. When the tumour is clinically localized, radical prostatectomy is the primary therapeutic modality, but when the disease is advanced, e.g., when there is a lymphatic metastatic spread, the effectiveness of the cure is severely reduced. In fact, 20% to 48% of men with prostate cancer leave the surgery room with positive tumour margins which directly correlates to biochemical recurrence (BCR) and cancer management [2,3]. The Prostate-Specific Membrane Antigen (PSMA), is a transmembrane protein that is overexpressed in 90–100% of PCa cells [4] and its expression increased markedly with tumour grade [5,6]. Due to its highly selective over-expression, PSMA is a reliable tissue marker for PCa and is considered an ideal target for tumour specific imaging and therapy. Urea-based inhibitors have been demonstrated to display a high affinity for PSMA [7]. Several small compounds for labelling PSMA have been developed and are currently investigated as imaging probes for Positron Emission Tomography (PET), with the ^68^Ga-labeled urea-based PSMA inhibitors being the most widely studied agents [8]. The 1st December 2020, the Food and Drug Administration approved ^68^Ga-PSMA-11 as PET agent for the diagnosis of prostate cancer metastasis and suspected prostate cancer recurrence [9]. However, the PET-based distribution of the PSMA expression in the lesion does not allow a reliable intraoperative identification because these lesions might be atypically located, too small, or morphologically unrecognizable [10]. On the other hand, fluorescence-guided surgery (FGS) has demonstrated to be an excellent option to allow a high spatial resolution discrimination between tumour and healthy tissues [11,12]. Compared to PET, optical guidance has a much superior spatial resolution and is detectable even down to the microscopic level [13]. Moreover, if the fluorophore is also a photosensitizer (PS) for photodynamic therapy, during surgery an adjuvant ablative therapy can be performed. Targeted photodynamic therapy (tPDT) consists of irradiating a photosensitive molecule at a specific wavelength that activates the photosensitizer. The activated photosensitizer transfers energy from light to molecular oxygen, to generate reactive oxygen species (ROS) [14]. Since ROS generation occurs in the areas of tissue that are exposed to light, the use of targeted photosensitizers may improve the selectivity and efficiency of this therapeutic strategy [15]. 

Recently, several approaches have been proposed for the intraoperative visualization and treatment of prostate cancer [15,16]. Most of them have been focused on small PSMA inhibitors coupled to the photosensitizer IRDye700DX. Pomper and co-workers proposed YC-9 as PSMA-targeted photodynamic therapy agent, demonstrating its efficacy both in vitro and in vivo after four PDT sessions [17]. Basilion and collaborators developed two PSMA-targeted PDT conjugates: PSMA-1-Pc413 and PSMA-1-IR700 showing strong tumour uptake and tumour growth inhibition [18]. They also demonstrated that the combination of FGS and tPDT reduced tumour recurrence and extended animal survival [19]. Other researchers proposed multimodal PSMA targeted agents composed by a photosensitizer and a radioactive isotope that were then used for imaging, surgical guidance and tPDT [20,21]. 

Benesova et al. identified PSMA-617 to improve the binding specificity and pharmacokinetics for both high imaging quality and efficient endoradiotherapy of recurrent PCa [22]. The most promising spacer selected from Benesova et al. based on the naphthylic and cyclohexyl linker structure has been adapted (Glu-NH-CO-NH-Lys-2-naphthyl-L-Ala-cyclohexane) to design a new PSMA inhibitor conjugated with IRDye700DX, suitable for the NIRF-guided surgery and photodynamic therapy. 

This work aims at synthesising for the first time a PSMA-617-targeted dual NIRF/PDT probe conceived using IRDye700DX as photosensitizer and FGS NIR dye, a glutamyl-urea-based PSMA inhibitor, and Glu-NH-CO-NH-Lys-2-naphthyl-L-Ala-cyclohexane moiety as spacer. The IRDye700DX confers to the molecule a bimodal feature for aiding surgeons in delineating positive tumour margins during surgery and triggering after surgery tPDT cytotoxicity on possible not resected tumour cells, while PSMA-617 confers superior specificity compared to the previous reported molecules. 

## 2. Results

### 2.1. Synthesis and Characterization of IRDye700DX-PSMA

**Figure 1:**
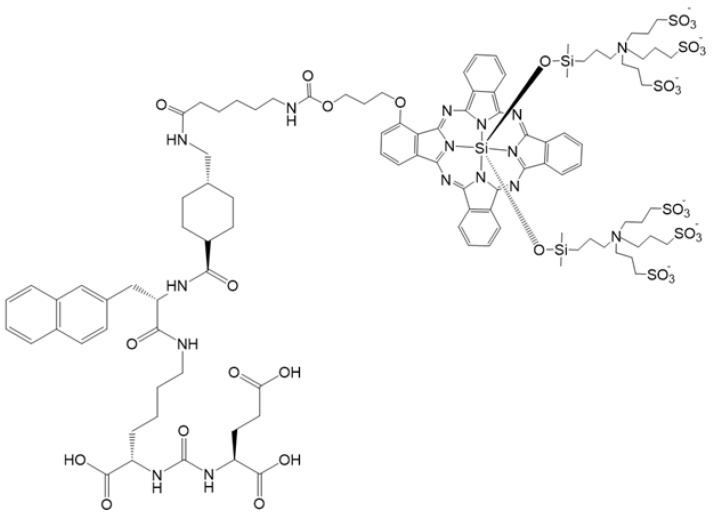
IRDye700DX-PSMA inhibitor molecular structure.

Figure 1 shows the chemical structure of the IRDye700DX-PSMA inhibitor having peptidomimetic glutamate-urea-lysine binding motif. To synthesize this conjugate, first, peptidomimetic Glu-NH-CO-NH-Lys-2-naphthyl-L-Ala-cyclohexane binding motif was synthesized by solid-phase peptide chemistry according to previously published method [23] and then reacted with IRDye700DX-NHS in a buffer phosphate 0.1 M, pH = 8. After purification by HPLC-UV-MS purity of the product was assessed by HPLC-UV-Vis-MS analysis to obtain a degree of purity of 100% at different wavelengths: 689 nm, 254 nm, 220 nm (Figure S2). IRDye700DX-PSMA has maximum absorbance (λ_abs_) at 689 nm and maximum emission (λ_em_) at 698 nm (Figure S3). IRDye700DX and IRDye700DX-PSMA shared the same absorbance and fluorescence maximum absorbance, thus demonstrating that the conjugation with the PSMA targeting moiety did not affect the fluorescence properties of the dye [24]. 

### 2.2. In Vitro Cellular Uptake of IRDye700DX-PSMA 

PSMA expression in LNCaP, PC3, PC3-PIP, and PC3-FLU prostate human cell lines was first examined by flow cytometry. As expected, PSMA expression was highest in PC3-PIP (MFI 1539 ± 355) followed by LNCaP (MFI 250 ± 111) and negligible in PC3 and PC3-FLU (MFI 5.5 ± 3 and 6.5 ± 7.5, respectively; Figure S4). This finding agreed with previous data reporting the number of PSMA receptors per cell [5,25]. 

**Figure 2:**
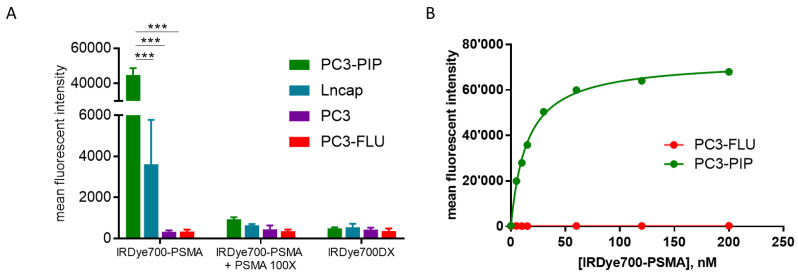
(**A**) Fluorescence emission of PC3-PIP (green), LNCaP (blue), PC3 (violet), PC3-FLU (red) cells following incubation with IRDye700DX-PSMA (500 nM), IRDye700DX (500 nM) and IRDye700DX-PSMA together with non-fluorescent peptide (50 μM). 2-way anova Bonferroni post hoc, *** *p* < 0.0001 n = 3; (**B**) Dose-dependent binding of IRDye700DX-PSMA to PC3-PIP (PSMA+ cell) and PC-3- FLU (PSMA-) n = 1.

The cellular uptake of IRDye700DX-PSMA towards the prostate tumour cells strictly followed the PSMA expression levels, with the highest avidity displayed by PC3-PIP cells (MFI 44812 ± 3883), while the uptake was much lower for LNCaP (MFI 3614 ± 2163), and almost negligible for the PSMA-negative cells PC3 (MFI 327 ± 68) and PC3-FLU (MFI 346 ± 91) (Figure 2A). Moreover, no uptake was observed when the probe was co-incubated with a 100x excess of the PSMA inhibitor and after the incubation of the untargeted IRDye700DX. Then, the uptake of IRDye700DX-PSMA was also evaluated upon incubation of increasing concentrations of the targeted dye with PC3-PIP or PC3-FLU cells (Figure 2B). IRDye700DX-PSMA displayed very high affinity (pK_d_ = 7.8 ± 0.1) to PSMA(+) PC3-PIP cells, whereas it did not bind to the PSMA(−) PC3-FLU cells. 

**Figure 3:**
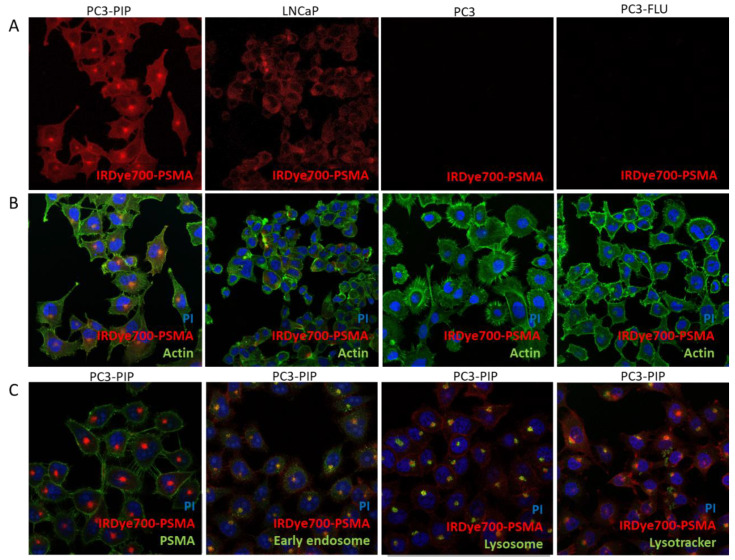
(**A**) Uptake and cellular distribution in PC3-PIP, LNCaP, PC3 and PC3-FLU of IRDye700DX-PSMA in red; (**B**) actin in green and nuclei in blue; (**C**) PC3-PIP cells incubated with IRDye700DX-PSMA in red and nuclei in blue, in green, respectively PSMA, early endosome, lysosome and lysotracker. Magnification 40×, PI 535–617 nm, AlexaFluor488 498–577 nm, IRDye700DX 650–750.

Confocal fluorescence microscopy confirmed the above-described results. IRDye700DX-PSMA signal was much stronger in PC3-PIP cells than in LNCaP, whereas no signal was detected in PC3-FLU and PC3 (Figure 3A,B). A different intracellular distribution of the dye was observed between LNCaP cells, which natively express PSMA, and PC3-PIP that are transfected to express PSMA receptors. A clear PSMA localization to the recycling endosome in perinuclear region was already observed in PC3-PIP but not in LNCaP cells [26]. In LNCaP, the endosomal compartments were found throughout the cytoplasm. Moreover, since for photodynamic therapy the cellular biodistribution is crucial, PC3-PIP cells were co-incubated with IRDye700DX-PSMA and different trackers of endolysosomal pathway (early endosome and lysosome; Figure 3C). The resulting images showed a co-distribution of the probe and endolysosomal markers in the recycling endosomes distributed in the perinuclear region, in agreement with previously reported data [26]. 

### 2.3. In Vivo Tumour Uptake and Biodistribution

**Figure 4:**
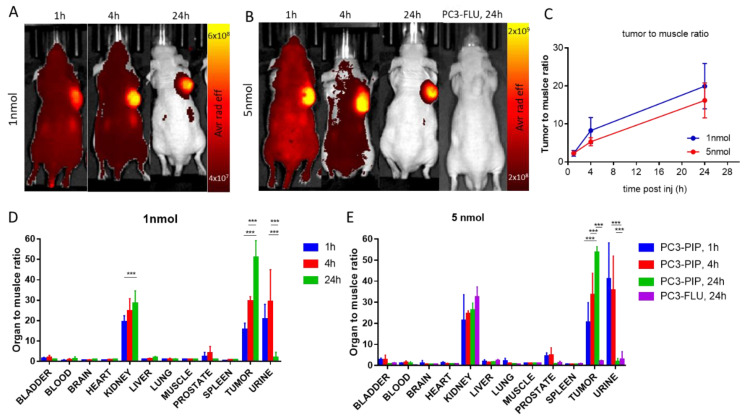
In vivo fluorescence imaging of PC3-PIP and PC3-FLU tumour bearing mice. (**A**) Representative in vivo optical images of PC3-PIP tumour bearing mice 1 h, 4 h and 24 h after administration of 1 nmol of IRDye700-PSMA (n = 4 group), λexc = 640 nm (30 nm bandwidth, λem = 720 nm. (20 nm bandwidth); (**B**) Representative in vivo optical images of PC3-PIP tumour bearing mice 1 h, 4 h and 24 h and PC3-FLU tumour bearing mice 24 h after administration of 5 nmol of IRDye700-PSMA (n = 4 group), λexc = 640 nm (30 nm bandwidth, λem = 720 nm. (20 nm bandwidth); (**C**) in vivo optical imaging signal ratio of tumour and muscle region at different timepoint after injection; (**D**) Ex vivo optical imaging of different organs after 1 nmol administration; (**E**) Ex vivo optical imaging of different organs after 5 nmol administration. *** *p* < 0.0001, 2-way ANOVA repeated measures followed by Bonferroni post hoc test.

NIRF imaging on mice bearing PC3-PIP xenografts showed a significant tumour uptake after the injection of both 1 and 5 nmol of IRDye700DX-PSMA at any time points (Figure 4A–C). The tumour-to-muscle ratio (TMR) did not change significantly between the two doses, with values of 20 ± 6 and 16.2 ± 4.6 24 h after the injection of 1 nmol and 5 nmol, respectively. The fluorescence signal measured on the explanted organs of the mice administered with the two doses, and normalized to the muscles, was reported in Figure 4D,E. The highest intensity was observed in tumour (TMR of 53.7 and 51.1 for 5 nmol and 1 nmol, respectively) and kidneys (kidney to muscle ratio of 26.4 and 28.6 for 5 nmol and 1 nmol, respectively) 24 h post-injection, thus confirming the good tumour uptake of the dye and its renal excretion. The fluorescence signal measured in both tumours and muscles was in good agreement with the injected doses (Figure S5), but while the control signal in the muscle decreased over time and reached the basal level 24 h post injection, the fluorescence in the tumour was almost constant, thus highlighting the effective targeting and accumulation of the dye in the lesion. 

The strong renal signal is probably due to the PSMA expression at kidney level [27].

Noteworthy, urine, healthy prostate, and bladder were not fluorescent at 24 h post injection. For 1 nmol urine-to-muscle ratio was 2.1 ± 2.5, prostate to muscle ratio was 0.95 ± 0.09 and bladder to muscle ratio was 1.03 ± 0.27. For 5 nmol urine to muscle ratio was 2.0 ± 1.4, prostate to muscle ratio was 0.83 ± 0.21 and bladder to muscle was 0.82 ± 0.11. These very low signals allow having no fluorescent background in surrounding prostate tumour during fluorescent guided surgery. 

The injection of 5 nmol of the PSMA-targeted dye in mice bearing the PSMA(-) PC3-FLU tumour resulted in a negligible fluorescence signal in the lesion and confirmed the excretion pathway of the probe (Figure 4E). In particular, the TMR in PC3-FLU was 2.2 that is 24 times lower compared to TMR observed in PC3-PIP under the same conditions. 

**Figure 5:**
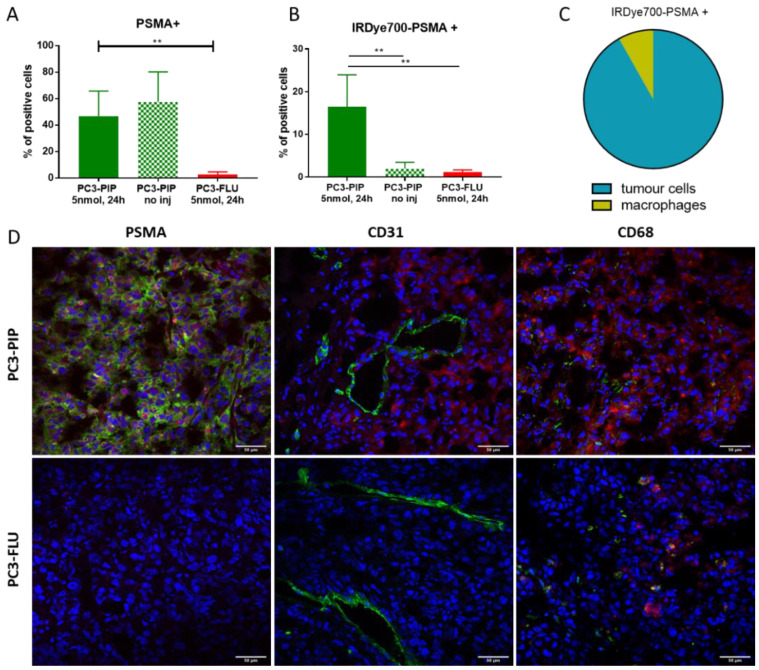
Ex vivo tumour characterization. (**A**) Flow cytometry data of % of PSMA positive cells in PC3-PIP and PC3-FLU tumour tissue. ** *p* < 0.01, 1-way ANOVA repeated measures followed by Bonferroni post hoc test. (**B**) Flow cytometry data of % IRDye700DX-PSMA positive cells in PC3-PIP and PC3-FLU tumour tissue. ** *p* < 0.01, 1-way ANOVA repeated measures followed by Bonferroni post hoc test (**C**) Flow cytometry data of IRDye700DX-PSMA cellular distribution in PC3-PIP tumours. (**D**) Confocal fluorescent images of PC3-PIP and PC3-FLU tumours with IRDye700DX-PSMA in red, nuclei in blue and in green PSMA, CD31 or CD68, respectively. Magnification 40×, PI (nuceli) 535–617 nm, AlexaFluor488 (PSMA, CD31, CD68) 498–577 nm, IRDye700DX 650–750.

PC3-PIP and PC3-FLU tumours explanted 24 h after the injection of 5 nmol of IRDye700DX-PSMA were further investigated by flow cytometry and confocal fluorescence microscopy (Figure 5). In PC3-PIP tumour, 50% of the cells were PSMA positive, while in PC3-FLU tumour the PSMA expression dropped down to 3% (Figure 5A). After Fluorescence Activated cell sorting (FACS), 16% of the PSMA(+) tumour cells were also IRDye700DX-PSMA positive, while in the PSMA(−) tumour only 1.9 % of the cells resulted positive to the dye. A similar low value (1.2%) was measured in the PSMA(+) lesions of mice not injected with the dye (Figure 5B). In PC3-PIP tumour, FACS analysis showed that the cells that internalized the probe were for the 7% macrophages and for the 88% tumour cells, confirming the high specificity of the probe for PSMA expressing cells (Figure 5C). 

Confocal fluorescence microscopy supported FACS data: the PSMA expression of PC3-PIP tumour was very clear, while no PSMA signal was detected in the PC3-FLU lesion. Signal from IRDye700DX-PSMA accumulated in PSMA(+) cells, but did not colocalize with the tumour endothelium (CD31 marker). The weak signal of IRDye700DX-PSMA detected in the PSMA(−) PC3-FLU tumour displayed a good colocalization with the CD68 macrophage marker (Figure 5D). 

**Figure 6:**
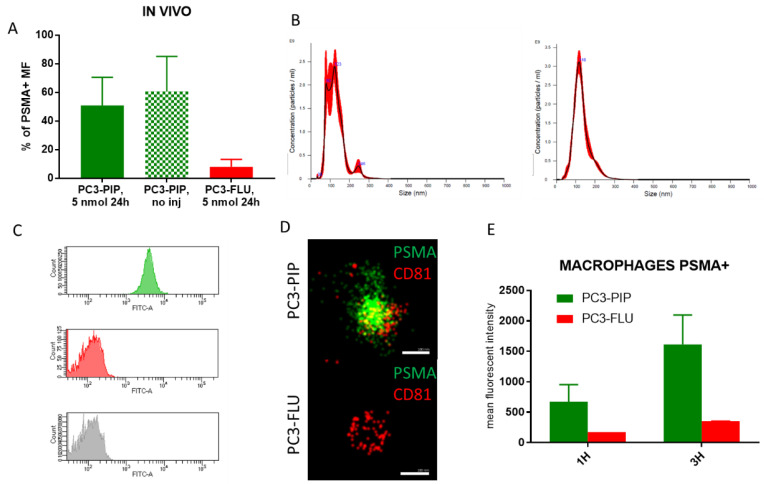
Extracellular Vesicles and macrophages interaction. (**A**) Flow cytometry data of % of PSMA positive macrophages in PC3-PIP and PC3-FLU tumour tissue; (**B**) representative graph of nanoparticles tracking analysis of EVs from PC3-PIP (left panel) and PC3-FLU (right panel) cells; (**C**) Representative flow cytometry data showing the expression of PSMA on the surface of PC3-PIP-EVs (green); PC3-FLU did not express PSMA (red). (**D**) Representative super resolution microscopy images of EVs from PC3-PIP (upper panel) and PC3-FLU (lower panel) showing the expression of PSMA (green) and CD81 (red). AlexaFluor488 (PSMA) 495–519 nm; AlexaFluor 647 (CD81) 650–655 nm. The scale bares are below each EV image (100 nm); (**E**) Flow cytometry data of PSMA in macrophages cell culture incubated with EVs from PC3-PIP or from PC3-FLU for 1 h and 3 h. As a proof of concept, a fluorescent guided surgery procedure, performed 24 h after the administration of 5 nmol of IRDye700DX-PSMA, was carried out in a mouse bearing PC3-PIP tumour (video in Supplementary Materials). The pictures taken before and after surgery (Figure 7) highlighted the good potential of the herein proposed probe for the visualization of the tumour margin and for verifying the complete resection of the lesion.

FACS data provided further important information on tumour microenvironment. We observed a different PSMA expression in Tumour-Associated-Macrophages (TAMs) between PC3-PIP and PC3-FLU tumours. 55% of TAMs associated with PC3-PIP tumour resulted to be PSMA positive, while in PC3-FLU tumour only the 8% of macrophages expressed PSMA (Figure 6A). As the fraction of TAMs that internalized the probe, 63% for PC3-PIP and 27% for PC3-FLU (Figure S6), was higher than the corresponding profile of PSMA expression (55% and 8%, respectively), the uptake of the IRDye700DX-PSMA probe by TAMs was partially aspecific.

To further investigate this finding, extracellular vesicles (EVs) were extracted from PC3-PIP and PC3-FLU cells. The presence of EVs were confirmed by NanoSight and by super resolution microscopy (Figure 6). No difference in dimension were detected by NanoSight (Figure 6B). Interestingly, EVs derived from PC3-PIP cells were PSMA+, while EVs from PC3-FLU were PSMA- (Figure 6C,D); on the contrary both types of EVs were positive for the classical exosomal marker CD81 (Figure 6D). Flow cytometry demonstrated that murine macrophages preincubated with EVs derived from PSMA+ cells expressed PSMA, while macrophages incubated with EV PSMA- did not (Figure 6E). These results support the view that EVs released by cancer cells are likely the responsible for the PSMA expression observed on the macrophages associated with PC3-PIP tumour. 

### 2.4. Photodynamic Therapeutic Effect

In vitro PDT experiments, conducted on PC3-PIP and PC3-FLU cells, showed a concentration-dependent decrease of cell viability (from 75% at 10 nM to 12% at 500 nM; Figure 8A) for the PSMA(+) cells. Under these conditions, the IC50 was 13.9 nM (Figure 8B). Importantly, the incubation of PC3-FLU cells with the maximum dose of the probe did not result in any cellular toxicity. The same result was obtained upon incubation with PC3-PIP cells without laser irradiation, using an excess of PSMA inhibitor, or using the untargeted dye, thus highlighting the high specificity of the targeted approach. 

In vivo, triggering the photodynamic effect produced a slight delay in the tumour progression in mice bearing PC3-PIP tumours (Figure 8C). In addition, the PDT treatment increased the median survival of the mice (Mantel-Cox test, *p* = 0.046, Figure 8D). 

## 3. Discussion

The development of efficient therapeutic strategies is fundamental for all cancers, especially for prostate cancer. From 1990s to 2000s a 53% decline in prostate cancer mortality has been observed thanks to PSA testing and advances in treatment. Despite that, 10% (estimated death 34,500 in USA for 2022) of men diagnosed with prostate cancer are expected to die in the next 5 years [1]. When the tumour is localized in prostate, surgery is the lead therapeutic option, but the absence of a distinct capsule around the prostate and intraoperative manipulations may complicate complete removal of cancer. The presence of positive margins after surgery is considered an adverse pathologic feature that requires additional therapeutic intervention [28]. The objective of the study was to develop a multi-approach theranostic molecule named IRDye700DX-PSMA, where the IRDye700DX unit may allow for both fluorescent guided surgery (FGS) and photodynamic therapy (PDT), and PSMA moiety is the chemical vector to direct the molecule to prostate cancer cells. 

Indeed, IRDye700DX-PSMA: (i) is highly specific in targeting prostate cancer cells demonstrating an uptake correlated with PSMA expression level (Figure 2 and Figure 3), (ii) allows NIR imaging with nanomolar dose up to 24 h post administration and, at the same time, shows a rapid clearance from PSMA-negative tissue (bladder included), (iii) may help surgeons to visualize negative tumour margins, iv) showed concentration dependent phototoxicity in PSMA+ cells but not in PSMA-, and v) delays tumour progression in photodynamic treated mice. 

Several studies have already demonstrated that PSMA is constitutively internalized via clathrin-coated pits to the recycling endosomal compartment (REC) [26,29,30]. IRDye700DX-PSMA follows the same pathway, demonstrating a colocalization with endolysosomal markers (Figure 3). This feature is particularly favourable for photodynamic therapy in PC3-PIP cell line because they have a perinuclear localization of REC. Indeed, different intracellular localization of photosensitizer can result in different cell fate outcomes [31,32,33,34].

IRDye700DX-PSMA showed a favourable biodistribution in mice, no liver or spleen uptake was observed at any timepoints, while the signal detected in kidneys and bladder confirms renal clearance. The kidney fluorescence is reported at any timepoint for both PC3-PIP and PC3-FLU tumour bearing mice (Figure 4). This is probably due to combined effect of renal excretion but also PSMA expression at kidney level [27,35,36]. Retention of small molecules targeting PSMA at kidney level is a well-known phenomenon [27,37]. Wester et al. reported a 20-times greater 68Ga-PSMA signal in kidney compared to LNCaP tumour [38]. For radionuclide therapy, radiation dosimetry at renal level is a critical factor for clinical translation of radiolabelled molecule [39]. IRDye700DX-PSMA does not show any dark toxicity, but on the other hand the toxicity induced by the molecule is generated only when the molecule is irradiated (Figure 8). The laser irradiation is localized in the tumour region, so the molecule results to be safe for kidney. Another favourable feature of IRDye700DX-PSMA is the loss of fluorescence in urine 24 h post injection (Figure 4). Considering the anatomical localization of prostate, this reduces possible background during FGS and toxicity in healthy tissue during irradiation. 

Furthermore, IRDye700DX-PSMA showed superior tumour-to-muscle ratio compared to most of the other small PSMA targeted molecules reported in literature so far. For example, the TMR value of IRDye700DX-PSMA is in the range of 16–20 1 h post administration versus 10 observed for 68Ga-PSMA-I&F [38]. A PSMA-targeted NIR agent (named OTL78) showed a tumour-to-muscle ranging from 19–25 after 2 h post administration [40], while IRDye700DX-PSMA showed a tumour-to-muscle of 30 at 4 h post administration and more than 50 at 24 h. TMR values ranging from 20 to 40 were reported 2 h post injection for a series of bimodal 111In-DOTA(GA)-IRDye700DX-PSMA probes [41]. A tumour-to-muscle ratio of 66.7 was reported by Heskamp and coworkers 2 h after 1 nmol administration of 111In-PSMA-N064 [20]. The great tumour specificity of IRDye700DX-PSMA is supported by the observation that 24 h post injection the fluorescence is 24-fold stronger in PC3-PIP tumour compared to PC3-FLU lesion. As comparison, for PSMA-1-IR700 4 h post injection, the signal in PC3-PIP tumour was more than 3.6-fold higher than PC3-FLU, and for PSMA-1-Pc413 the difference was even lower (about 2-fold) [18]. 64Cu-LC-Pyro showed a 4-fold selective accumulation in PC3-PIP compared to PC3-FLU after 17 h post administration [42]. 

**Figure 7:**
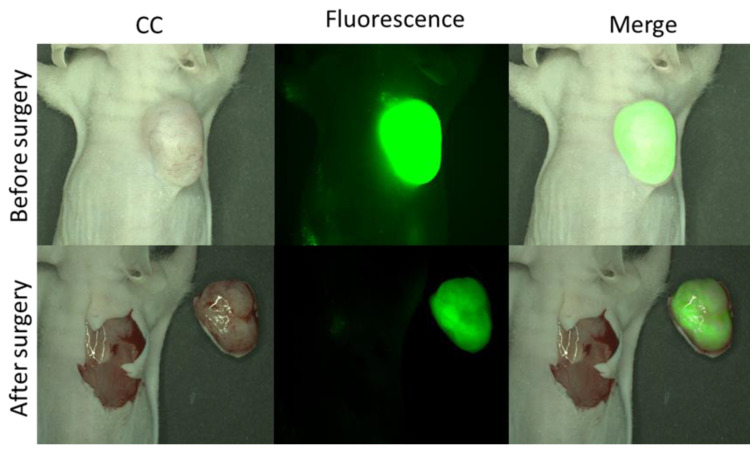
Representative images of Fluorescence Guided Surgery on PC3-PIP tumour bearing mice administered with 5 nmol of IRDye700DX-PSMA 24 h before (excitation/emission filters: (Ex: 615/665 nm, Em: 695/770 nm).

**Figure 8:**
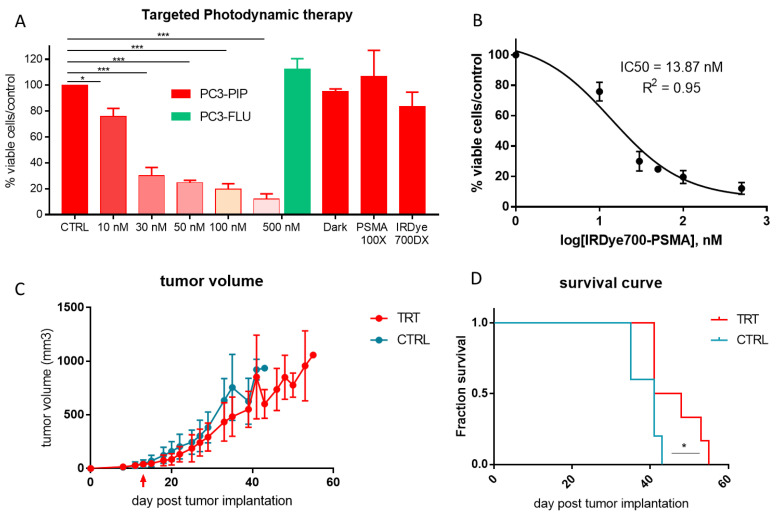
Photodynamic therapeutic efficacy in vitro and in vivo. (**A**) MTT vitality test of IRDye700DX-PSMA at different concentration (range 10–500 nM) on PC3-PIP (red) and PC3-FLU (green) 1 h post irradiation; * *p* < 0.05, *** *p* < 0.0001 1-way ANOVA repeated measures followed by Bonferroni post hoc test. (**B**) IC50 value of vitality test of PC3-PIP treated and irradiated. (**C**) Tumour volume measured via MRI, red arrow indicates the treatment. (**D**) Survival curve of PC3-PIP tumour bearing mice after treatment, Mantel-Cox test, * *p* < 0.05.

A great advantage for future clinical application of IRDye700DX-PSMA is that the tumour signal remains stable up to 24 h as observed in fluorescence biodistribution (Figure 4) and in FGS proof of concept (Figure 7). Accordingly, confocal images and flow cytometry data showed an exceptional colocalization of PSMA receptor and IRDye700DX-PSMA (Figure 5) confirming the specificity of binding. 

An important collateral finding of this work is the observation that the tumour associated macrophages of the PC3-PIP PSMA+ tumour displayed PSMA expression, thus contributing to the tumour uptake of the targeted agent. In vitro experiments clearly supported the hypothesis that the PSMA expression on macrophages was promoted in vivo by extracellular vesicles secreted by the PSMA+ tumour cells. The relevance of extracellular vesicles in prostate cancer has been extensively investigated. Vesicles released from prostate cancer cells carry molecular information about the disease [43,44]. It has been recently reported that PSMA positive plasma-derived vesicles are increased in patients with advanced metastatic prostate cancer [45]. Moreover, EVs play an important role in cross-communication between prostate tumour cells and microenvironment influencing tumour progression and metastatic spreading [46,47,48]. EV’s natural cargo involved various bioactive molecules, including nucleic acids, proteins, lipids, transcriptional factors, and RNA-binding proteins [49,50]. Here, we observed that 100% of EVs released by PC3-PIP expressed PSMA on the EV surface. Moreover, 55% of macrophages in PC3-PIP tumour expressed PSMA, while the expression of PC3-FLU tumour was much lower (8%) (Figure 6). Furthermore, it was sufficient to incubate murine macrophages with PSMA+ EVs for 1 h to induce a significant increase in the expression level of PSMA. Since in PC3-PIP tumour the 7% of total cells positive to IRDye700-PSMA were macrophages, their additional expression of the target would have a beneficial effect on the overall performance of the targeted molecule. Most likely, this finding may be extended to many other kinds of tumours or other pathologies, thus opening new diagnostic and therapeutic strategies for improving targeted treatments.

In recent years photodynamic therapy (PDT) has been deeply investigated thanks to the availability of new efficient photosensitizers [51,52]. Targeted photodynamic therapy improves the efficacy of the treatment and reduce the toxicity. Although in vitro this is widely demonstrated, in vivo experimental results are often lacking or show only a delay in tumour growth after repeated administration and irradiation cycles [17,18]. To improve the outcomes, Rijpkema et al. irradiated mice with increasing laser fluence rate (in the range of 50–150 J/cm^2^), with 150 J/cm^2^ that showed the best results, but with the appearance of side effects [21]. 

All these data taken together suggest that PDT alone is not enough to kill the tumours completely [53]. This is a known Achilles heel for clinical translatability of targeted photodynamic therapy in general. Tumour recurrence is often reported in clinical trials [54] and it is supposed due to insufficient light penetration but also due to PDT resistant tumours [55]. The idea suggested to overcome this drawback is to combine different photosensitiser to target different tumour compartments, or to combine PDT with chemotherapy [56]. 

A limitation in the present study is the tumour model. Indeed, all the study has been performed in a subcutaneous tumour model that is suboptimal to simulate fluorescent guided surgery and tPDT experiment. However, it should be considered that an orthotopic model was not suitable for surgery. 

## 4. Materials and Methods

### 4.1. Synthesis and Characterization of IRDye700DX-PSMA

All chemicals, reagents, and solvents for the synthesis were purchased from Sigma Aldrich (St. Louis, MO, USA), Merck (Darmstadt, Germany) and Iris Biotech (Marktredwitz, Germany). IRDye^®^ 700DX NHS Ester was obtained from LI-COR, Inc. (Lincoln, NE). Analytical and preparative HPLC−MS was carried out on a Waters AutoPurification system (3100 Mass Detector, 2545 Pump Gradient Module, 2767 Sample Manager, and 2998 PDA detector). 

The peptidomimetic PSMA binding motif glutamate-urea-lysine binding motif (Glu-NH-CO-NH-Lys-2-naphthyl-L-Ala-cyclohexane) was synthesized by solid-phase peptide chemistry according to previously published methods (23). The PSMA binding motif (1 mg, 1.5 μmol), was conjugated to IRDye700DX-NHS (1.9 mg, 1 μmol) in buffer phosphate 0.1 M, pH = 8 The reaction was stirred for 2 h at room temperature followed by preparative HPLC-MS. The details of the preparation and characterization of IRDye700DX-PSMA are reported in the Supplementary Materials.

### 4.2. Cell Culture

The PSMA positive (PSMA+) PC3-PIP and PSMA negative (PSMA−) PC3-FLU were kindly provided by Prof Martin G. Pomper (Johns Hopkins Medical School, Baltimore, MD, USA) [57]. PC3 and LNCaP cell line were purchased from ATCC. LNCaP and PC3 cell lines were cultured until confluence using Ham’s F12 (Euroclone) for PC3 or RPMI-1640 (Euroclone) medium for LNCaP, PC3-PIP and PC3-FLU supplemented with glutamine (2 mM), 10% foetal bovine serum (FBS, Sigma-Aldrich, St. Louis, MO, USA) and penicillin/streptomycin antibiotics (10,000 IU/mL penicillin, 10,000 IU/mL streptomycin, Corning Cellgro, Manassas, VA, USA). PC3-PIP and PC3-FLU cell lines were grown under 20 μg/mL of puromycin to maintain PSMA expression.

### 4.3. PSMA Expression by Flow Cytometry

Cells were seeded into a T75 flask and allowed to form a monolayer over 48 h. After gentle rinsing with PBS, the cell dissociation non-enzymatic solution (Sigma) was added, and the flask left in the humidified incubator (37 °C) for 15 min. The cells were then collected in medium, centrifuged (1100 rpm, 5 min), counted and split into different test tubes (5 × 10^5^ cells/tube per 100 µL). All the samples were centrifuged, and the supernatant removed. The different cell lines were then incubated for 30 min at 4 °C with 98 μL of a solution containing BSA 5% and 2 μL of PSMA Antibody, anti-human, Vio^®^ Bright FITC, REAfinity™ or REA Control Antibody (S), human IgG1, Vio^®^ Bright FITC, REAfinity (Miltenyi Biotec, Bologna Italy). After washing in PBS 0.2% bovine serum albumin and centrifugation (1100 rpm, 5 min) cell pellets were resuspended in 100 μL of buffer solution. The samples, after adding propidium iodide to evaluate the cell viability, were analysed on a BDFACSVerse^®^ instrument (BD, New Jersey, USA) using laser 488 nm with emission filter 530/40 nm. 1 × 10^4^ live cells/tube were acquired. Samples were analysed with BDFACSSuite and with FlowJO10.5.3 softwares. In vitro binding. The affinity of IRDye700DX-PSMA was tested following the same protocol applied for PSMA expression, incubating for 1 h at 37 °C the LNCaP, PC3, PC3-PIP and PC3-FLU cell lines with 500 nM of IRDye700DX-PSMA, IRDye700DX, IRDye700DX-PSMA together with a non-fluorescent peptide (PSMA-617, 50 μM). The samples were analysed on FACSVerse^®^ instruments (BD, New Jersey, USA) using laser 663 nm with emission filter 750-long pass.

For IRDye700DX-PSMA binding affinity, PC3-PIP and PC3-FLU cells were seeded into a 12-well plate (100,000 cell/well) and incubated with increasing concentrations of IRDyeDX700-PSMA (in the range between 5 and 200 nM) for 1 h at 37 °C. After two rinsing with cold PBS, cells were detached with non-enzymatic solution. All the samples were centrifuged (1100 rpm, 5 min). Cell pellets were resuspended in 100 μL of buffer solution. The samples, after adding propidium iodide to evaluate the cell viability, were analysed on FACSVerse^®^ instruments (BD, New Jersey, USA) using laser 633 nm with emission filter 750-long pass. The binding affinity (Kd) was calculated using a plot of percent mean fluorescent intensity versus concentration using GraphPad Prism 6.

### 4.4. Confocal Images

For confocal images experiments, PC3-PIP, LNCaP, PC3-FLU and PC3 cells were seeded into μ slide 8-well (ibidi GmbH, 82166 Gräfelfing, Germany) at a cell-seeding density of 5 × 10^4^ cells per well. After 24 h, cells were incubated with 500 nM of IRDye700DX-PSMA for 1 h at 37 °C. LysoTracker Green DND-26 (Invitrogen) at a final concentration of 100 nM was added 2 min before the end of incubation for lysosome staining. After the incubation, cells were washed twice with cold PBS, and fixed with 4% paraformaldehyde solution in PBS for 10 min. For immunofluorescence studies PC3-PIP, LNCaP, PC3-FLU and PC3 cells were permeabilized in 0.1% Triton X 100 in PBS for 10 min, saturated in 5% normal goat serum (NGS) in PBS for 1 h and incubated with a primary antibody for 45 min at RT. The following antibodies were used: mouse monoclonal anti-EEA1 (sc-365652 Santacruz, Dallas, TX, USA) for early endosome, mouse monoclonal anti-LAMP1 (sc-20011 Santacruz, Dallas, TX, USA) for lysosome and anti-PSMA. Primary antibodies were detected with anti-mouse Alexa Fluor 488 (Molecular Probes, Invitrogen) used at 1:500 dilution for 1 h. Cells were also stained using a FITC phalloidin solution (P5284 Sigma) at a concentration of 0.2 µg/mL for 30 min. For cell nuclei, cells were treated with RNase A solution (final concentration 0.5 µg/mL) and stained with propidium iodide (PI) for 30 min. Observations were conducted under a confocal microscopy (Leica TCS SP5 imaging system) equipped with an argon ion. IRDye700-PSMA was visualized using 633 nm laser, Phalloidin and Alexa Fluor 488 secondary antibody were imaged using 488 nm laser, PI was imaged using 561 nm laser.

### 4.5. In Vivo Biodistribution and Tumour Uptake

Athymic Nude-Foxn1nu mice were provided by Envigo RMS, S.r.l., Udine Italy. All the procedures involving the animals are conducted according to the national and international laws on experimental animals (L.D. 26/2014; Directive 2010/63/EU). Mice were subcutaneously implanted with 2 × 10^6^ PC3-PIP or PC3-FLU cells in the right shoulder. Imaging experiments were performed approximately 21 days after cells implantation.

PC3-PIP tumour bearing mice (n = 5/group) were injected with two doses (1 and 5 nmol) of IRDye700DX-PSMA and imaged at 1 h, 4 h and 24 h post injection. PC3-FLU tumour bearing mice (n = 5/group) were administered with 5 nmol of IRDye700DX-PSMA and imaged at 24 h post injection. The optical imaging experiments were performed on IVIS Instrument SPECTRUM (Perkin Elmer), equipped with a CCD camera. The optimal filter pair were λexc = 640 nm, λem = 720 nm. After imaging, mice were sacrificed, and organs harvested to detect the fluorescence signal.

### 4.6. Ex Vivo Tumour Analysis

PC3-PIP and PC3-FLU tumours explanted from mice treated with 5 nmol of IRDye700DX-PSMA after optical imaging experiments were divided in two pieces: one was processed for flow cytometry detection while the other piece was fixed for confocal microscopy analysis. For flow cytometry tissue processing was performed as already reported [58]. Briefly, tumours were kept in MACS tissue storage solution (Miltenyi Biotec) until processing. Samples were minced into small pieces and enzymatically digested with collagenase I (final concentration 0.25–1 U ml-1; Sigma-Aldrich) for 45 min at 37 °C with agitation (220 rpm). After digestion, samples were filtered with a 70-μm cell strainer (BD Biosciences, San Jose, CA, USA), centrifuged (1400 rpm, 10 min) and incubated in erythrocyte lysing buffer (155 mM NH4Cl, 15.8 mM Na2CO3, 1 mM EDTA, pH 7.3) for 10 min at room temperature. After washing in RPMI-1640 with 10% FBS, 1 × 10^6^ cells were collected, re-suspended in PBS, and treated with Fc-receptor blocker (anti-CD16/CD32 antibody, ThermoFisher Scientific). Samples were then stained with anti-human PSMA antibody, anti-mouse CD45 antibody VioGreen, anti-mouse F4/80 antibody PE-Vio 770 (Miltenyi Biotec) for 30 min at 4 °C. Samples were acquired on BDFACSVerse and cell populations analysed with FlowJO10.5.3

For confocal images, tumours were placed in optical cutting temperature (OCT) compound and snap-frozen in liquid nitrogen vapor. Sections at 5 μm thickness were cut and fixed with 4% PAF solution in PBS for 15 min. For immunofluorescence experiments tissue sections were permeabilized in 0.1% Triton X 100 in PBS for 10 min, saturated in 5% normal goat serum (NGS) in PBS for 1 h and incubated with a rabbit polyclonal anti-CD68 antibody (ab125212 Abcam, Cambridge, UK), anti-PSMA (130-118-336 Miltenyi Biotec), or rat monoclonal anti-CD31 (557355 BD Pharmingen, Milano Italy) for 45 min at RT. An anti-rabbit and anti-rat Alexa Fluor 488 antibody (Molecular Probes, Invitrogen. Eugene, OR, USA) at 1:500 dilution for 1 h was used to detect the primary antibodies. For cell nuclei, cells were treated with RNase A solution (final concentration 0.5µg/mL) and stained with propidium iodide (PI) for 30 min. Observations were conducted under a confocal microscopy (Leica TCS SP5 imaging system) as stated above.

### 4.7. Extracellular Vesicles Isolation and Characterization

PC3-PIP and PC3-FLU cells were starved overnight (16 h) in RPMI FBS free medium when they reached 80% confluency. Then, the supernatant was collected and centrifuged for 10 min at 300× *g* to remove cell debris. The supernatant was filtered with 0.2 μm filter and it was subsequently ultracentrifuged for 2 h at 100,000× *g*, at 4 °C. The EV pellet was resuspended in RPMI supplemented with 1% DMSO. The EV suspension was then stored at −80 °C until further use.

After EV isolation, the EV concentration was measured by Nanosight NS300 (Malvern Instruments Ltd., Malvern, UK) equipped with a 488 nm laser module. Samples were diluted 1:200 in physiologic solution. For each sample, 3 videos of 30 s at camera level 15 and threshold 5 were captured using a syringe pump 30. All the samples were characterised with NTA 3.2 Analytical software. The NTA settings were kept constant between samples.

Super-resolution microscopy pictures of EVs were obtained using a temperature-controlled Nanoimager S Mark II microscope from ONI (Oxford Nanoimaging, Oxford, UK) equipped with a 100x, 1.4NA oil immersion objective, an XYZ closed-loop piezo 736 stage, and 405 nm/150 mW, 473 nm/1 W, 560 nm/1 W, 640 nm/1 W lasers. Two-channel (647 and 488) dSTORM data (3000 frames per channel) were acquired sequentially at 30 Hertz in total reflection fluorescence (TIRF) mode [50]. For sample preparation, we followed the manufacturer’s protocol using EV profiler Kit (ONI). Samples were labelled with the anti-CD81-647 antibody (from the kit) and PSMA-FITC antibody (130-118-336 Miltenyi Biotec). Before each imaging session, beads slide calibration was performed to align fluorescent channels, achieving a channel mapping precision smaller than 12 nm. Images were taken in dSTORM mode acquired sequentially in total reflection fluorescence (TIRF) mode. Single-molecule data was filtered using NimOS (Version 1.18.3, ONI, Oxford, UK) based on the point spread function shape, photon count and localisation precision to minimise background noise and remove low-precision and non-specific co-localisation. All pictures were analysed using algorithms developed by ONI via their CODI website platform (https://alto.codi.bio/, 11 February 2022). The drift correction pipeline version 0.2.3 was used.

Flow cytometric analysis was performed on EVs adsorbed onto surfactant-free white aldehyde/sulfate latex beads 4% *w*/*v*, 5µm diameter (A37306, Invitrogen, Waltham, Massachusetts, USA) using a FACS Celesta instrument (Becton Dickinson, Franklyn Lake, NJ, USA). Five μL of aldehyde/sulfate latex beads were incubated with 6 × 10^9^ EVs overnight rotating at 4 °C. The second day 1 mL of 100 mM glycine dissolved in PBS was add to each sample and incubated on rotor for 30 min. After the incubation, the samples were spin down at 2700× *g* for 3 min at room temperature. The samples were washed twice with 0.5% PBS BSA and after that, the samples were resuspended in 200 μL. To each sample, 1 μL of anti-PSMA FITC (130-118-336 Miltenyi Biotec) and REA CTL (130-113-443 Miltenyi Biotec) were added, and the mix was incubated rotating in dark for 30 min. After two washing steps the samples were resuspended in final volume 200 μL for the acquisition.

### 4.8. Macrophage Isolation

Murine bone marrow-derived macrophages were isolated as recently reported by Garello et al. [59]. Briefly, immediately after sacrifice femurs and tibias were excised, cleaned from the connective tissue and muscles, and the bone epiphyses were cut away. Bone cavities were flushed with DMEM/F-12 (Dulbecco’s Modified Eagle Medium/Nutrient Mixture F-12, EuroClone, Milano, Italy) medium, until appearing white. Cell clump and tissue debris were removed by filtering the suspension through a 70 µm cell strainer (BD Biosciences Pharmingen, San Diego, CA, USA). The filtered suspension was centrifuged (10 min, 500× *g*), the supernatant discarded, and the pellet of bone marrow-derived cells was re-suspended in 4 mL of DMEM/F-12 supplemented with 10% (*v*/*v*) FBS, 12.5 mM L-glutamine, 100 U/mL penicillin and 100 μg/mL streptomycin. Bone marrow-derived cells were seeded on sterile pre-treated glass coverslips placed into 6 cm Petri dishes. Cells were incubated for 7 days at 37 °C, 5% CO_2_, with 20 ng/mL of macrophage colony-stimulating factor (M-CSF; Merck KGaA, Darmstadt, Germany) to obtain adherent non-polarized-M0 macrophages. After 7 days, macrophages (approximately 250 000 macrophages for 6 cm Petri dishes) were incubated for 1 h or 3 h with about 1 × 10^10^ EVs (50 000 EVs for target cell). After the incubation macrophages were detached by scraper and stained as for tumour tissue for flow cytometry analysis.

### 4.9. Fluorescence Guided Surgery

Fluorescence Guided Surgery (FGS) simulation was performed on mice bearing PC3-PIP tumour (n = 2). To this purpose, 5 nmol of IRDye700-PSMA per mouse was administered intravenously. 24 h p.i. mice were sacrificed. To evaluate tumour accumulation of the targeting probe, the mice were imaged under an AxioZoom.v16 planar microscope, equipped with a HXP 200C illuminator (Mercury short arc lamp) and dedicated excitation/emission filters (Ex: 615/665 nm, Em: 695/770 nm). Prostate tumours were then removed.

### 4.10. In Vitro Targeted Photodynamic Therapy

5 × 10^4^ PC3-PIP and PC3-FLU cells were allowed to adhere overnight to 96-well plates. The cells were incubated with increasing concentration of IRDye700DX-PSMA (from 10 nM to 500 nM) for 1 h at 37 °C. As negative controls (i) PC3-FLU cells were incubated with 500 nM of IRDye700DX-PSMA, (ii) PC3-PIP cells were incubated with 500 nM of IRDye700DX-PSMA and 50 μM of PSMA-617, (iii) PC3-PIP cells were incubated with IRDye700DX. The plates are washed two times with PBS and then irradiated for 4 min at room temperature in 96-well plate using a 500 mW lamp (λ = 635 nm) with an energy flow rate of 30 J/cm^2^. One hour post irradiation, cell viability is quantified by using MTT (3-(4,5-dimethyl-2-thiazole)-2,5-diphenil-2H-tetrazolium bromide; thiazole blue) assay. To test dark toxicity, PC3-PIP cells are incubated with 500 nM of IRDye700DX-PSMA for 1 h. Three independent experiments are performed.

### 4.11. In Vivo Targeted Photodynamic Therapy

Athymic Nude-Foxn1nu mice are provided by Envigo RMS, S.r.l., Udine Italia. Mice were subcutaneously implanted with 2 × 10^6^ PC3-PIP cells in the right shoulder. Treatment was performed 13 days after cells implantation with a tumour volume of 43 ± 30 mm^3^. 5 nmol of IRDye700DX-PSMA were intravenously administered 24 h before irradiation. Tumour region was irradiated for 20 min at room temperature using a 500 mW lamp (λ = 635 nm) for a total light energy used of 190 J/cm^2^. Three times a week, tumours were acquired using a 7 T Bruker Pharmascan (Bruker Biospin, Ettlingen, Germany) scanner equipped with a 30 mm 1H quadrature coil. Before imaging, mice were anesthetized by intramuscular injection of a combination of 20 mg/kg tiletamine/zolazepam (Zoletil 100; Virbac, Milan, Italy) and 5 mg/kg xylazine (Rompun; Bayer, Milan, Italy). T2 weighted (T2w) axial images were acquired with the following parameters: Echo time (TE) = 33 ms, Repetition Time (TR) = 2500 ms, Number of Averages = 2, matrix size = 256 × 256, Field of View (FOV) = 35 × 35 mm, slice thickness = 0.8 mm, acquisition time = 2 min and 40 sec. Tumour volume were calculated as the sum of tumour areas measured by manual outlining on every slice of the T2– weighted datasets multiplied by the slice thickness.

## 5. Conclusions

In conclusion, here we propose the PSMA targeted IRDye700DX-PSMA agent for dual synergistic fluorescent guided surgery and photodynamic therapy applications. The molecule was successfully tested in terms of selectivity for PSMA+ cells, in vivo PSMA tumour uptake and biodistribution, and FGS and photodynamic experiment. The herein proposed agent showed high-affinity with excellent PSMA-specific tumour uptake. These results are very encouraging and hold on further preclinical testing even combining PDT with others therapeutic strategies.

## Data Availability

Not applicable.

## Supplementary Materials

The following supporting information can be downloaded at: https://www.mdpi.com/article/10.3390/ijms232112878/s1, Figure S1: Analytical HPLC of PSMA-617 on Atlantis dC18 column RP, 5 µm, 4.6 × 150 mm, H2O-TFA 0.1%, CH3CN- TFA 0.1% on HPLC-UV-Vis-MS system, A: Chromatograms recorded at wavelengths 220 nm. B: mass spectrum ESI(+) MS m/z relative to the peak at 13.48 min.; Figure S2: Analytical HPLC of IRDye700DX-PSMA on Atlantis dC18 column RP, 5 µm, 4.6 × 150 mm, ammonium acetate 10 mM in water and acetonitrile on HPLC-UV-Vis-MS system, A: Chromatograms recorded at different wavelengths: 689 nm and 225 nm. B: mass spectrum ESI(-) MS m/z relative to the peak at 6.8 min.; Figure S3: Fluorescence excitation and emission spectra of IRDye700DX-PSMA; Figure S4: PSMA expression measurement via Flow cytometry in PC3-PIP, LNCaP, PC3-FLU and PC3 cell line; Video S1: targeted fluorescent guided surgery in PC3-PIP tumor bearing mice administered with 5 nmol of IRDye700DX-PSMA.
