# Supplementary material for: A Novel PSMA-Targeted Probe for NIRF-Guided Surgery and Photodynamic Therapy: Synthesis and Preclinical Validation
Source: Int J Mol Sci. 2022 Oct 25;23(21):12878. doi: 10.3390/ijms232112878 (PMC9657290; doi:10.3390/ijms232112878)
Supplement: Supplementary file 1 [file ijms-23-12878-s001.zip › supplementary material ijms.pdf]

SUPPLEMENTARY MATERIAL

A novel PSMA-targeted probe for NIRF-guided surgery and photodynamic therapy: synthesis and preclinical validation

Martina Capozza [1], Rachele Stefania [1], Valentina Dinatale[1], Valeria Bitonto [1], Laura Conti [2], Cristina Grange [2], Renata Skovronova [3] and Enzo Terreno [1,*]

1. Molecular & Preclinical Imaging Centers, Department of Molecular Biotechnology and Health Sciences, University of Torino, 10126 Torino, Italy
2. Molecular Biotechnology Center, Department of Medical Science, University of Torino, Torino, Italy
3. Molecular Biotechnology Center, Department of Molecular Biotechnology and Health Sciences, University of Torino, 10126 Torino, Italy

* Correspondence: enzo.terreno@unito.it.

Martina Capozza and Rachele Stefania equally contributed as first authors

Synthesis and characterization of IRDye700DX-PSMA

All chemicals, reagents, and solvents for the synthesis were purchased from Sigma Aldrich, Merck and Iris Biotech. IRDye® 700DX NHS Ester was obtained from LI-COR, Inc. (Lincoln, NE). Analytical and preparative HPLC–MS was carried out on a Waters AutoPurification system (3100 Mass Detector, 2545 Pump Gradient Module, 2767 Sample Manager, and 2998 PDA detector).

The peptidomimetic glutamate-urea-lysine binding motif (Glu-NH-CO-NH-Lys-2-naphthyl-L-Ala-cyclohexane) was synthesized by solid-phase peptide chemistry according to previously published methods on 0.1 mmol scale. Cleavage from the solid support and amino acid side chain deprotections were performed simultaneously by adding a solution of TFA:TIPS:water (95:2.5:2.5) for 2 hours, then filtered to remove the resin. Cold diethyl ether was then added to precipitate the crude compound. The compound pellet was dissolved in 1 mL of water and purified by HPLC-MS, the separation was performed on an Atlantis dC18 OBD Prep Column, 100Å, 5 μm, 19 mm X 100 mm, Eluent: A: 0.1% TFA in water; B : 0.1% TFA in acetonitrile, Gradient: 10%B→30%B over 6 min, 30%B→40%B over 11 min , Flow rate: 20mL/min. After purification, the product was frozen and lyophilized to obtain 30 mg of a solid with white color (yield 46 %). Analytical HPLC-UV-MS was carried out using an Atlantis RP-C18, 3.5 μm, 4.6 mm X 150 mm column, Eluent: A : 0.1% TFA in water; B : 0.1% TFA in acetonitrile, Gradient: 90%B→10%B over 2min, 10%B→30%B over 8 min, 30%B→40%B over 20 min, 40%B→100%B over 6 min, Flow rate: 1mL/min. The chromatogram revealed at 220 nm showed one major peak corresponding to a degree of purity of 92% (retention time 12.8 min). ESI+MS m/z relative to the peak at 12.8 min (calcd. for C33H45N5O9): [M+H]+ 656.3 (obsd.), 656.7 (calcd.) (Figure S1).

PSMA binding motif (1 mg, 1.5 μmol), was conjugated to IRDye700DX-NHS (1.9 mg, 1 μmol) in buffer phosphate 0.1 M, pH = 8 The reaction was stirred for 2 h at room temperature followed by preparative HPLC-MS, the separation was performed on an Atlantis dC18 OBD Prep Column, 100Å, 5 μm, 19 mm X 100 mm, Eluent: A : ammonium acetate 10 mM in water; B : acetonitrile, Gradient: 10%B→50%B over 12 min, Flow rate: 20mL/min. After purification, the product was frozen and lyophilized to obtain 1,2 mg of a solid with dark blue color (yield 49 %). Analytical HPLC was carried out using an Atlantis RP-C18 column, 5 μm, 4.6 mm x 150 mm, ammonium acetate 10mM (solvent A) and acetonitrile (solvent B); Gradient: 20%B→50%B over 20min, 50%B→100%B over 10 min, Flow rate: 1mL/min. The chromatogram (Figure S1) showed one peak corresponding to a degree of purity of 94,4 %, based on the chromatographic peak area revealed at 225 nm and at 689 nm (retention time 6.8 min). ESI(-) MS m/z relative to the peak at 6.8 min calculated for calculated for C103H140N16O33S6Si32- [M-2H]2- 1202.9, found 1202.8, C103H139N16O33S6Si33- [M-3H]3- 801.6, found 801.0, C103H138N16O33S6Si34- [M-4H]4- 601.2, found 600.3.

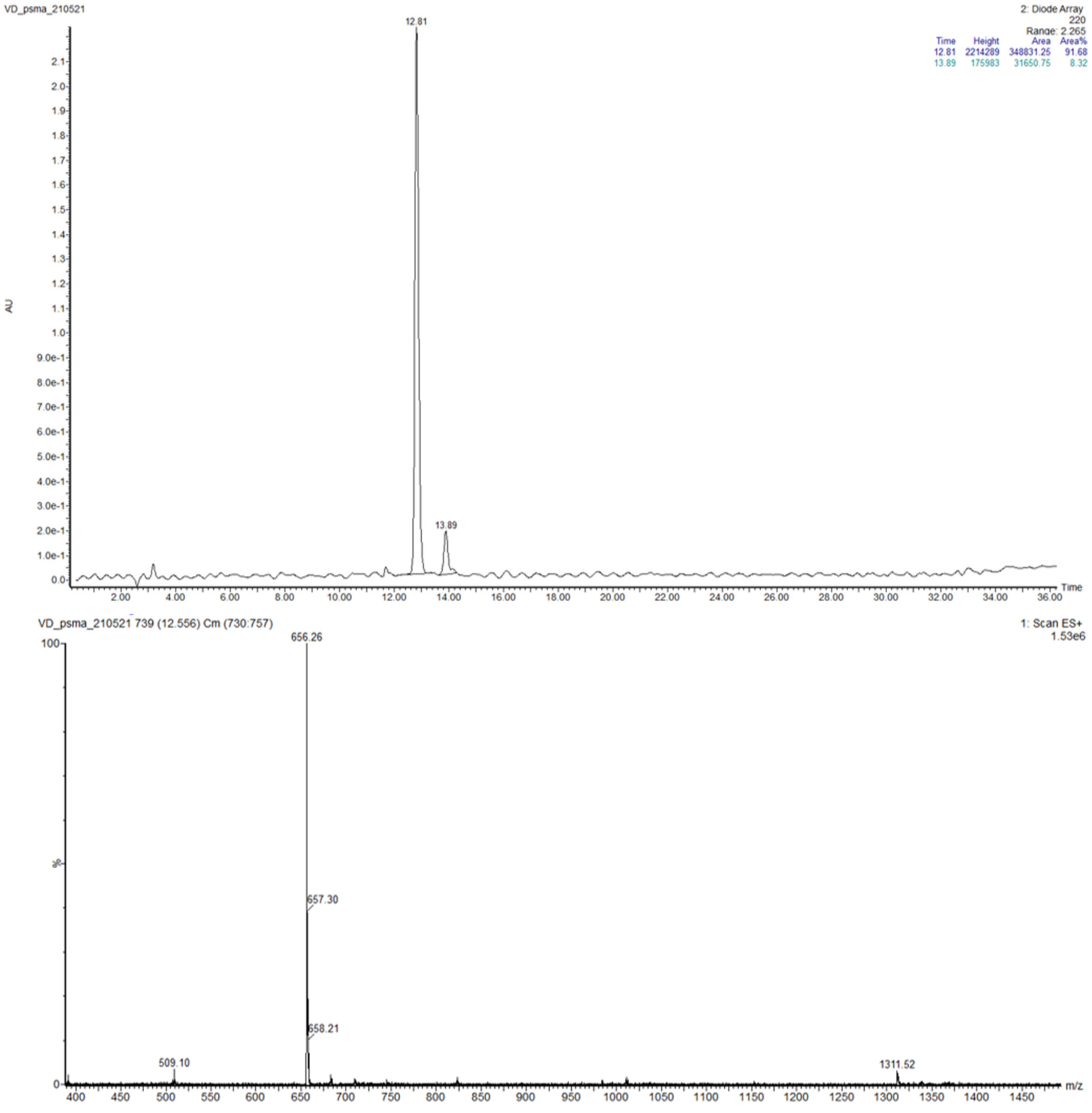

Figure S1. Analytical HPLC of PSMA-617 on Atlantis dC18 column RP, 5 µm, 4.6 mm x 150 mm, H2O-TFA 0,1%, CH3CN-TFA 0,1% on HPLC-UV-Vis-MS system, A: Chromatograms recorded at wavelengths 220 nm. B: mass spectrum ESI(+) MS m/z relative to the peak at 13.48 min.

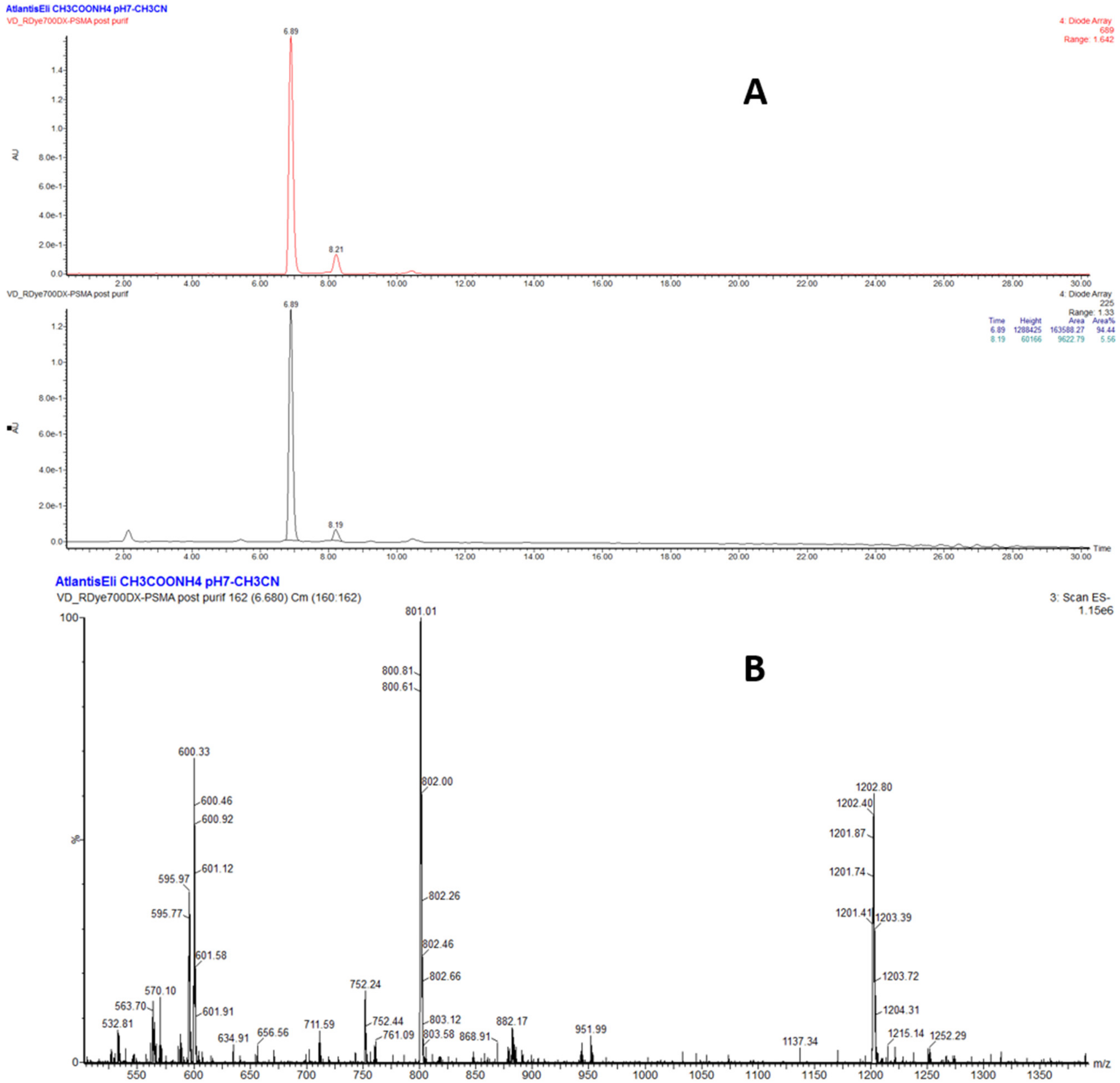

Figure S2. Analytical HPLC of IRDye700DX-PSMA on Atlantis dC18 column RP, 5 µm, 4.6 mm x 150 mm, ammonium acetate 10mM in water and acetonitrile on HPLC-UV-Vis-MS system, A: Chromatograms recorded at different wavelengths: 689 nm and 225 nm. B: mass spectrum ESI(-) MS m/z relative to the peak at 6.8 min.

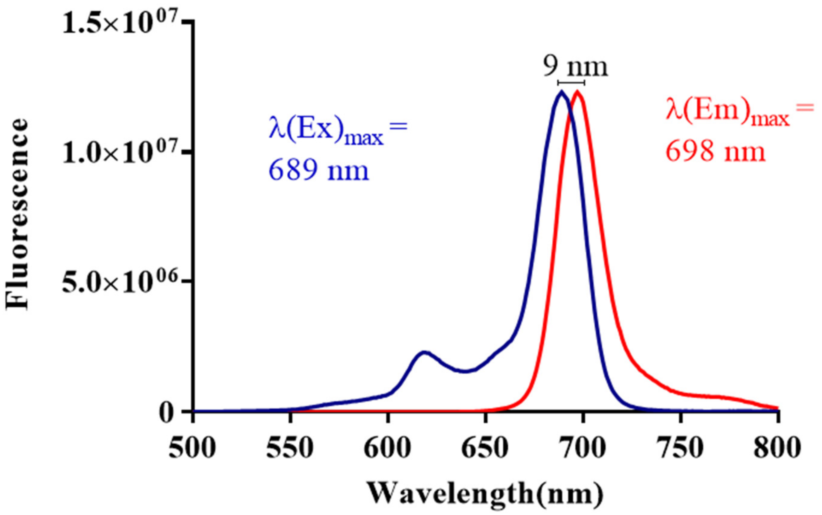

Figure S3. Fluorescence excitation and emission spectra of IRDye700DX-PSMA

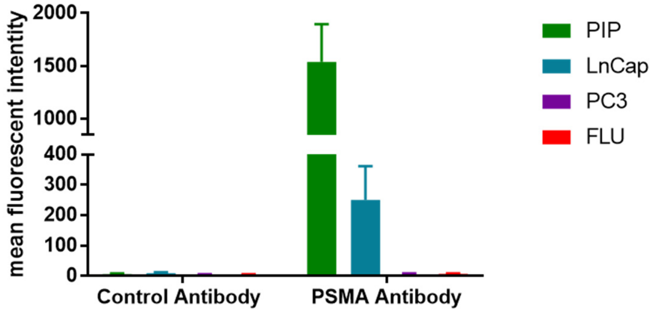

Figure S4. PSMA expression measurement via Flow cytometry in PC3-PIP, LNCaP, PC3-FLU and PC3 cell line

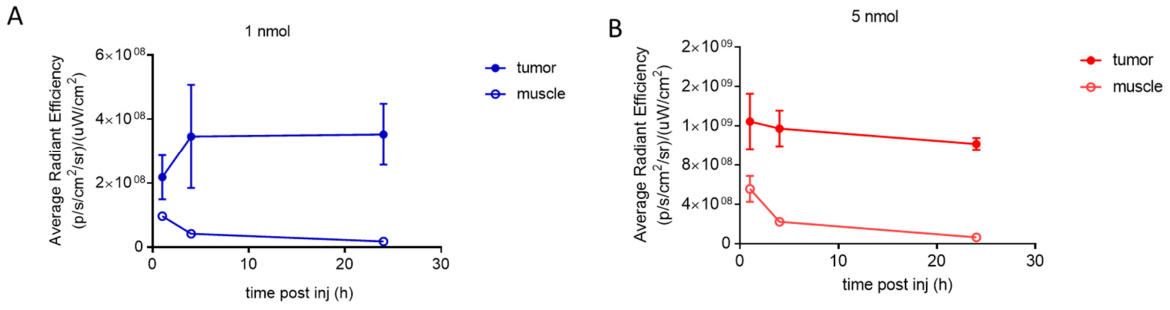

Figure S5. Fluorescent signal calculated from in vivo optical imaging in tumor and muscle region post 1 nmol (A) and 5nmol (B) of IRDye700DX-PSMA.

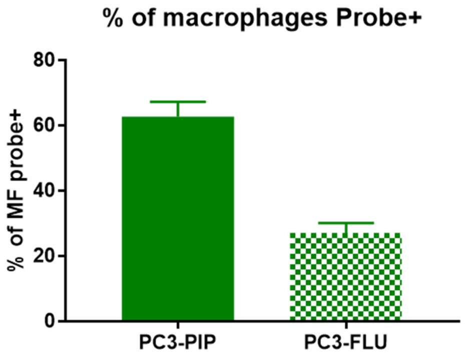

Figure S6. Ex vivo tumour characterization. Flow cytometry data of % of macrophages that taken IRDye700DX-PSMA up to the total macrophages population in the tumor.
